# Proteomic Analysis of the Dysferlin Protein Complex Unveils Its Importance for Sarcolemmal Maintenance and Integrity

**DOI:** 10.1371/journal.pone.0013854

**Published:** 2010-11-05

**Authors:** Antoine de Morrée, Paul J. Hensbergen, Herman H. H. B. M. van Haagen, Irina Dragan, André M. Deelder, Peter A. C. ’t Hoen, Rune R. Frants, Silvère M. van der Maarel

**Affiliations:** 1 Center for Human Genetics, Leiden University Medical Center, Leiden, The Netherlands; 2 Biomolecular Mass Spectrometry Unit, Department of Parasitology, Leiden University Medical Center, Leiden, The Netherlands; Brigham and Women's Hospital, Harvard Medical School, United States of America

## Abstract

Dysferlin is critical for repair of muscle membranes after damage. Mutations in dysferlin lead to a progressive muscular dystrophy. Recent studies suggest additional roles for dysferlin. We set out to study dysferlin's protein-protein interactions to obtain comprehensive knowledge of dysferlin functionalities in a myogenic context. We developed a robust and reproducible method to isolate dysferlin protein complexes from cells and tissue. We analyzed the composition of these complexes in cultured myoblasts, myotubes and skeletal muscle tissue by mass spectrometry and subsequently inferred potential protein functions through bioinformatics analyses. Our data confirm previously reported interactions and support a function for dysferlin as a vesicle trafficking protein. In addition novel potential functionalities were uncovered, including phagocytosis and focal adhesion. Our data reveal that the dysferlin protein complex has a dynamic composition as a function of myogenic differentiation. We provide additional experimental evidence and show dysferlin localization to, and interaction with the focal adhesion protein vinculin at the sarcolemma. Finally, our studies reveal evidence for cross-talk between dysferlin and its protein family member myoferlin. Together our analyses show that dysferlin is not only a membrane repair protein but also important for muscle membrane maintenance and integrity.

## Introduction

Dysferlin (DYSF, MIM*603009) is a 230 kDa large transmembrane protein highly expressed in striated muscle and to a lesser extent in other tissues, including monocytes, syncytiotrophoblast, endothelium, brain, pancreas, and kidney.[1] Dysferlin is found intracellularly on vesicles and at the plasma membrane. Upon laser-inflicted membrane damage dysferlin rapidly accumulates at the site of the lesion in a calcium dependent manner, and participates in patch-fusion repair. In the absence of dysferlin the membrane tear is not adequately repaired and the myofiber will undergo necrosis.[2]

Mutations in the dysferlin gene cause a spectrum of adult-onset progressive muscular dystrophies including Limb Girdle Muscular Dystrophy type 2B (LGMD2B, MIM#253601), Myoshi Myopathy (MM, MIM#254130), and Distal Anterior Compartment Myopathy (DACM, MIM#606768), commonly referred to as dysferlinopathies.[3]–[5] There is no clear genotype-phenotype correlation and the ∼150 described mutations cover the complete open reading frame. (www.dmd.nl/dysf) It is therefore unclear how defects in the DYSF gene cause muscular dystrophy. It has been suggested that the skeletal muscle membrane is continuously subject to mechanical wear and tear, and that the dysferlin deficiency phenotype results from inefficient membrane repair in response to continued membrane damage.[6] Dysferlin knockout mice develop a phenotype similar to the dysferlinopathies.[2]

Dysferlin contains seven C2 domains, two DysF domains and a C-terminal transmembrane domain. C2 domains are calcium sensitive phospholipid binding domains, as was also shown for the first C2 domain (C2A) of dysferlin [7], and are thought to be important for regulating dysferlin trafficking. These domains have also been shown to interact with proteins.[8] The function of the DysF domain remains unclear.[9] Dysferlin (also known as ferlin1-like 1, FER1L1) belongs to the family of ferlin-like proteins that includes otoferlin (FER1L2, MIM *603681), myoferlin (FER1L3, MIM *604603), FER1L4, FER1L5 and FER1L6. The family is named after ferlin, a *Caenorhabditis elegans* gene that when mutated causes infertility [10] and muscle dysfunction [11]. Ferlin is essential for the fusion of vesicles with the cell membrane.[10] Mutations in otoferlin cause an autosomal recessive form of congenital deafness (DFNB9, MIM #601071).[12] Otoferlin is expressed in hair cells in the inner ear [13], and participates in the trafficking of synaptosomal vesicles.[14] Myoferlin is like dysferlin strongly expressed in muscle.[15] It has been suggested that myoferlin might be able to replace dysferlin as a potential strategy for therapy of dysferlinopathies.[15], [16] The exact function of myoferlin remains unclear. FER1L4, FER1L5 and FER1L6 proteins have not yet been characterized.

While the role of dysferlin in membrane repair is well established, it is less clear how the protein is regulated. Likely it requires binding partners that aid in vesicle nucleation, localization, targeting and recycling. To date only few of such cofactors have been identified, yet they yielded important insight into dysferlin function.

MG53 (TRIM72, MIM *613288) is a redox sensor that participates in vesicle nucleation.[17] It can interact with dysferlin.[18] Together with the dysferlin interacting protein caveolin 3 it is involved in the trafficking of dysferlin to and from the sarcolemma.[18]–[20] In addition, annexins A1 and A2 can bind dysferlin in a calcium-dependent manner.[21] These proteins are membrane fusogens that participate in lysosome exocytosis.[22] They are thought to aid in dysferlin vesicle targeting. The cysteine protease Calpain 3 (CAPN3, MIM *114240) co-immunoprecipitates with dysferlin in skeletal muscle.[23], [24] It is hypothesized to play a role in cytoskeleton remodeling, [24], [25] and is predicted to remodel cytoskeletal structures to allow for patch fusion repair.[24], [26], [27] Finally, AHNAK (MIM *103390) is found on enlargosomes which have been implicated in membrane enlargement and repair.[28]–[30] AHNAK interacts with dysferlin in skeletal muscle, an interaction that is regulated by calpain 3 activity.[24] These protein interactions have thus yielded some information on dysferlin function in membrane repair.

Recent data however, suggests that dysferlin is more than a membrane repair protein. It has been shown to be involved in cytokine [31] and chemokine [32] secretion and associates with developing t-tubules.[33] In fertilized sea urchin embryo's dysferlin participates in extracellular ATP signaling.[34] Moreover, a defect in membrane repair cannot fully explain the patient's phenotype, which has been reported to include renal [35] and cardiac failure [36]. In addition, dysferlin is considered to be a very dynamic protein, which is found in the cytosol in myoblasts and regenerating myofibers, but shows a more prominent membranous localization in mature skeletal muscle tissue.[31] It was shown that dysferlin trafficking and endocytosis depend on its direct interaction with caveolin 3.[19], [20], [37]

We hypothesized that a comprehensive overview of dysferlin function can be inferred from large-scale proteomics analysis of dysferlin protein complexes. Dissecting complex protein structures requires highly specific affinity binders. We have previously described heavy chain antibody fragments (HCAb) that specifically recognize dysferlin in mice and humans.[23] These HCAb make for exceptional tools to dissect the dysferlin protein complex due to their strong performance in immunoprecipitation (IP) experiments. By immunoprecipitation of endogenous dysferlin from human skeletal muscle followed by mass-spectrometry and western blotting we could previously identify calpain 3 and AHNAK to interact with dysferlin.[8], [23]

In this study we compared dysferlin protein complexes from proliferating myoblasts, differentiated myotubes and mature skeletal muscle tissue. We show that 1) we can efficiently and reproducibly immunoprecipitate dysferlin protein complexes from these sources, 2) bioinformatics analyses of mass spectrometry identified proteins confirm a role for dysferlin as a vesicle protein, and 3) such analyses reveal new layers of dysferlin function which we substantiated by further exploring interactions with myoferlin, and focal adhesion components.

## Results

### Robust and reproducible isolation of dysferlin protein complexes

**Figure 1:**
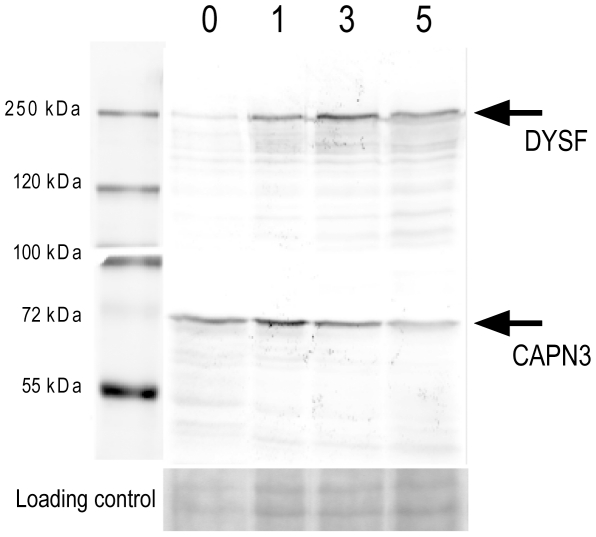
Dysferlin is upregulated during myogenic differentiation in IM2 cells. IM2 cells were differentiated and harvested at day 0, 1, 3 and 5. Protein lysates were probed on western blot for Dysferlin (upper panel) and Calpain 3 (lower panel).

As dysferlin expression is reported to increase with myogenic differentiation we hypothesized that the composition of its protein complex might also change during this process. Therefore we aimed to isolate dysferlin protein complexes from different stages of myogenic differentiation. We used the IM2 cell model [38] as a source for dysferlin protein complexes to establish the optimal immunoprecipitation conditions. This cell line can be grown indefinitely under permissive conditions, and switched to myogenic differentiation by serum deprivation. The IM2 cell model performs more consistently than the C2C12 cell line in terms of myogenic capacity and differentiates within a shorter time-span. We first established by western blotting that IM2 cells express dysferlin (Figure 1). Dysferlin expression increases with differentiation, in line with previous data from C2C12 myoblast cells [39]. Calpain 3, an established partner in the dysferlin protein complex is also expressed in these cells (Figure 1).

**Figure 2:**
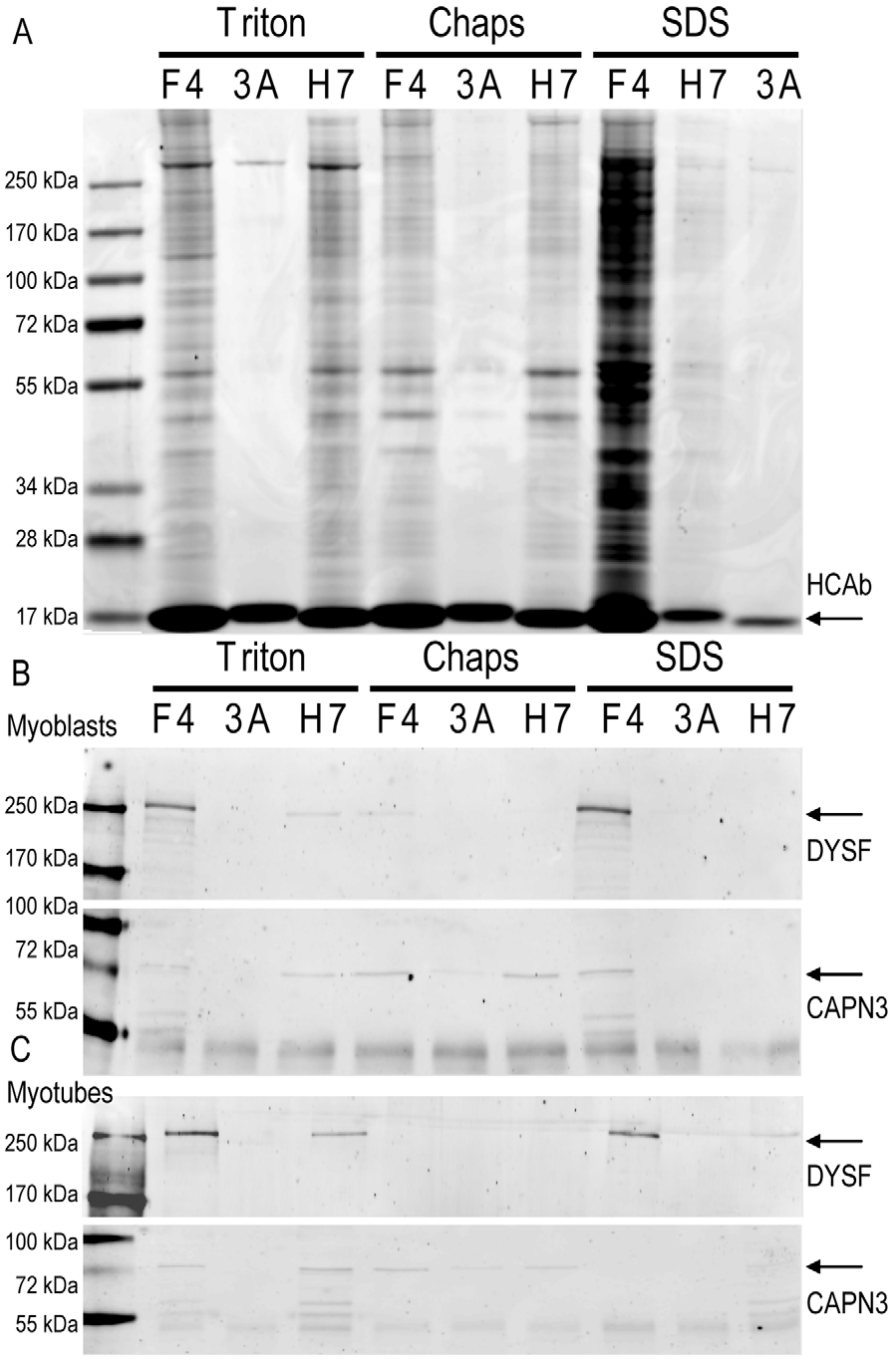
Reproducible dysferlin immunoprecipitation under different conditions. IM2 myoblasts and myotubes were lysed in three different buffers and subjected to a HCAb Dysferlin immuno precipitation protocol. F4 and H7 are specific for Dysferlin while 3A is a non-specfic control HCAb. A) Coomassie stained gel of immunoprecipitation fractions from myoblasts. B) western blot for Dysferlin and Calpain 3 corresponding to the gel in A. C) A similar western blot on myotubes IP fractions (corresponding gel not shown).

We previously reported two dysferlin specific llama heavy chain antibody fragments (HCAb) that can specifically immunoprecipitate dysferlin from human skeletal muscle.[23] We adapted the immunoprecipitation method for cultured cells by testing different buffer conditions (Figure 2). We used the two reported dysferlin HCAb fragments, F4 and H7 [23], and compared their performance to a non-specific HCAb (3A), selected against amyloid-β [40] and which does not recognize dysferlin. We selected three different lysis buffers for our experiments. The first buffer is of low ionic strength and contains 0.2% Triton. Triton is a comparatively mild non-denaturing non-ionic detergent. The second buffer is of high ionic strength containing SDS and TEA and has a strong solubilizing capacity. At the used concentration (0.1%) SDS acts as a partially denaturing detergent that is efficient at breaking low-afinity protein-protein interactions. The third buffer contains CHAPS (0.15%) and is of intermediate strength. CHAPS is a nondenaturing zwitterionic detergent that is useful for solubilizing membrane proteins. The CHAPS buffer has been used previously to precipitate dysferlin protein complexes from muscle tissue with HCAb [23] and with conventional antibodies [37].

Both anti-dysferlin HCAb can precipitate protein complexes in a mild buffer with low Triton concentration (Figure 2A) whereas the non-specific HCAb 3A fails to immunoprecipitate dysferlin or other proteins. The zwitter-ionic buffer (CHAPS) results in a reduction of IP efficiency, consistent with the increased stringency of the buffer. Again, the non-specific HCAb does not immunoprecipitate detectable protein amounts. Finally we lysed the cells in SDS buffer (0.1%). The SDS is predicted to disfavor transient interactions and should therefore result in a decreased IP of dysferlin complexes. As predicted, only a limited amount of proteins coimmunoprecipitate with H7 in SDS buffer. F4 however, immunoprecipitates an increased number of proteins. A concomitant western blot for dysferlin and its interaction partner calpain 3 confirmed the Coomassie blue stained gels (Figures 2B (myoblasts) and 2C (myotubes)). From this we conclude that the HCAb can be used in different buffer conditions, and that H7 is the HCAb of choice for qualitative IP experiments, while F4 can be used for confirmation experiments. Moreover, the Triton buffer yields the highest level of protein complexes and is therefore preferred as lysis buffer. Because it is more difficult to homogenize tissue than cultured cells, we decided to use CHAPS buffer for skeletal muscle tissue as we have done previously [23].

### Dysferlin interactions in myoblasts, myotubes, and skeletal muscle tissue

**Figure 3:**
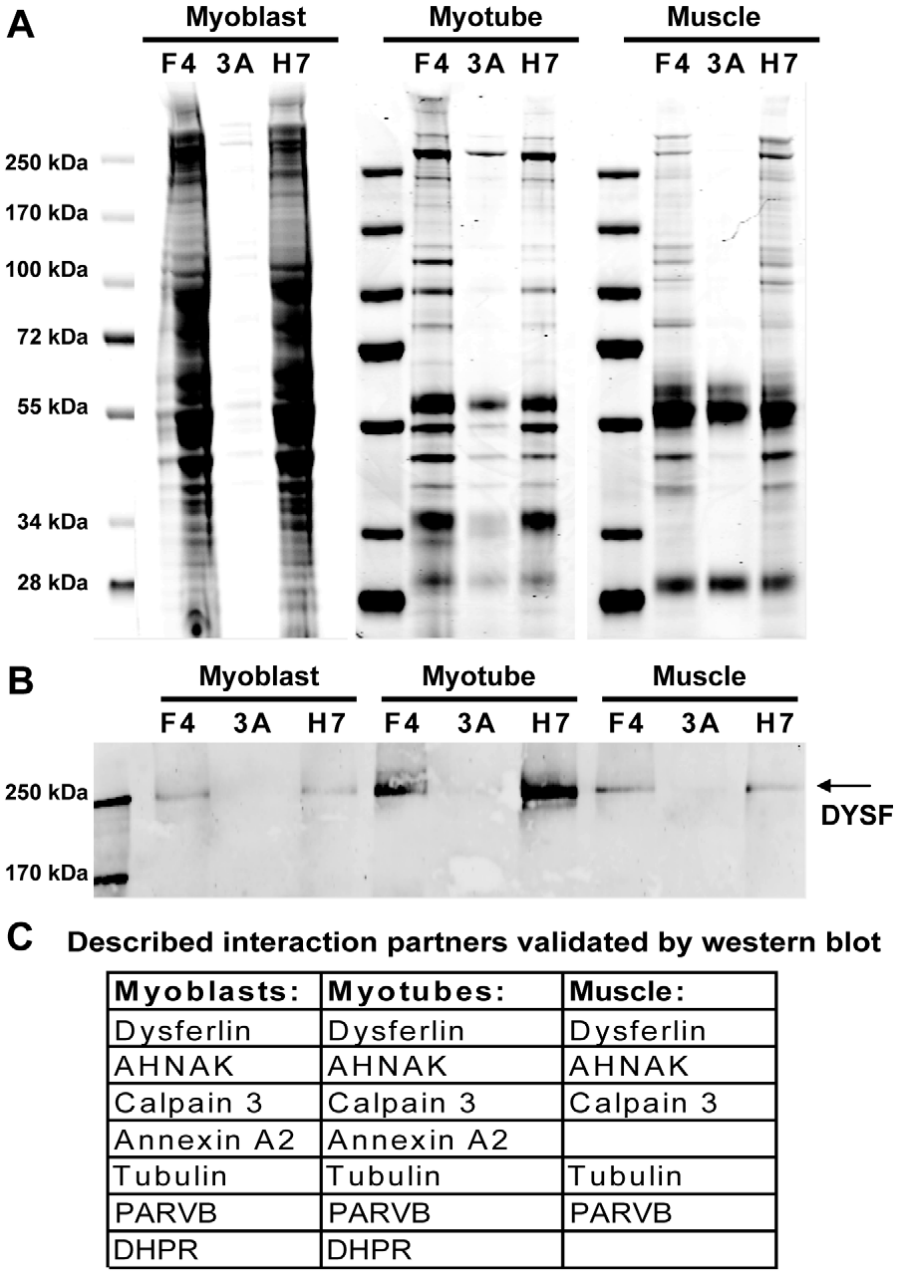
Immunoprecipitation of dysferlin from different myogenic sources is highly reproducible. A) Coomassie blue stained gel of IP fractions from IM2 myoblasts, myotubes and skeletal muscle tissue. IP samples of two dysferlin specific HCAb (F4 and H7) yield highly similar staining patterns, contrary to a non-specific HCAb (3A). B) a concomitant western blot for dysferlin confirms the IP. C) All protein bands stained in A), lanes H7, were excised, in-gel trypsin digested and analyzed by mass spectrometry. Many described interaction partners of dysferlin were identified by western blot (Figure S2) and thus confirmed.

We continued with preparing lysates for IM2 myoblasts, IM2 myotubes at 5 days post-differentiation, when spontaneous contraction was observed, and human skeletal muscle tissue. After cell lysis in Triton buffer or tissue homogenization in CHAPS buffer, dysferlin protein complexes were immunoprecipitated from these lysates using HCAb H7, separated on SDS-PAGE gels and stained with Coomassie-blue for further mass spectrometry analysis (Figure 3a). We monitored the IP procedure with western blot (Figure S1). The IP was confirmed by western blot with a conventional antibody specific for dysferlin. (Figures S1 and 3b) With western blotting we could confirm six of the described interactions (TUBA[41] (Figure S1) CAPN3[23], AHNAK[8] (both in Figure S2), PARVB[42], DHPR[43], ANXA2[21] (data not shown), summarized in Figure 3c. Mass spectrometry analysis confirmed the presence of one additional reported dysferlin interaction partner (MG53[18] Table S1), but also resulted in the identification of many new putative binding partners (complete lists in table S1). Skeletal muscle cells contain a large amount of sarcomeric proteins, and components of the dystrophin glycoprotein complex (DGC). We did not observe the core DGC components in the IP fractions, and the amount of sarcomeric proteins is low, consistent with the fact that the HCAb specifically recognize and immunoprecipitate dysferlin. We conclude that the IP method efficiently and reproducibly enriches for dysferlin protein complexes.

**Figure 4:**
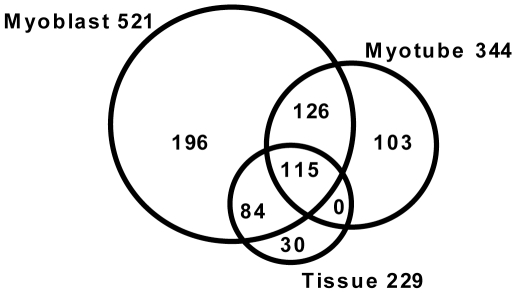
Venn diagram showing the overlap of dysferlin interaction partners in myoblast, myotubes and skeletal muscle tissue. All Coomassie stained bands from the H7 IP shown in Figure 3A were excised, in-gel digested and analyzed by mass spectrometry. 115 proteins are consistently identified in all three protein sources.

In our IP data set for myoblasts we identified 521 proteins annotated in online databases, 344 proteins in myotubes and 229 proteins in skeletal muscle tissue (full lists in supplemental tables S1 and S2). 115 proteins are shared by all datasets (Figure 4). These proteins we therefore consider a core-set of most likely dysferlin interacting proteins, or representative of functionalities of dysferlin that do not change during differentiation. As mentioned before, the tissue IP was performed under more stringent conditions. Interestingly, 85% of the proteins identified in the tissue IP were also identified in the cultured cells. This underlines the reproducibility of the IP experiment, and indicates a relatively low number of proteins that nonspecifically co-immunoprecipitate with dysferlin. An unexpected finding was that there are no proteins shared by IP from tissue and myotube alone. This might be explained by the more stringent conditions used for tissue homogenization.

### Potential interaction between dysferlin and myoferlin

**Figure 5:**
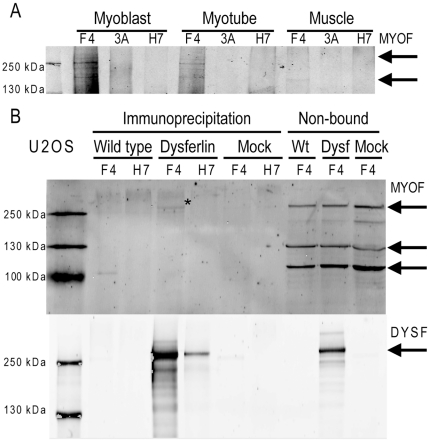
Myoferlin and dysferlin may be present in the same protein complex. A) Dysferlin IP fractions were stained in western blot with a myoferlin specific antibody, to verify that full-length dysferlin is co-immunoprecipitated with dysferlin, and not with the control HCAb 3A. B) U2OS cells, which express endogenous myoferlin but not dysferlin, were transfected with dysferlin cDNA or empty vector (mock), and both untransfected (wild-type) and transfected cells were lysed and subjected to an IP experiment with F4 and H7. IP and non-bound fractions were analyzed on western blot for dysferlin (lower panel) and myoferlin (upper panel). F4 and H7 can only immunoprecipitate dysferlin from dysferlin transfected cells (lower panel), consistent with the absence of endogenous dysferlin expression. Myoferlin is not immunoprecipitated from wild-type cells, though it can be detected in the non-bound fractions. However, myoferlin is immunoprecipitated by F4 from dysferlin expressing U2OS cells (band marked by asterisk).

We were surprised to consistently identify the dysferlin homolog myoferlin to co-immunoprecipitate with dysferlin (Mascot protein score of 300, 12 unique peptides in myoblast IP sample). We do not think this occurs due to cross reactivity of our antibodies. Myoferlin shares 68% amino acid sequence similarity with dysferlin.[15] However, the dysferlin HCAb were previously shown not to cross-react with recombinant myoferlin.[23] Moreover, the dysferlin HCAb failed to immunoprecipitate proteins from dysferlin negative myoblasts and myotubes, as judged from Coomassie stained gels and western blots for dysferlin and myoferlin (not shown). Finally, when we used western blotting to confirm the presence of full-length myoferlin in the H7 and F4 IP fractions (Figure 5), we observed that it is absent in the control HCAb 3A fractions. Highest levels of myoferlin are detected in the myoblast IP fractions. We therefore think that dysferlin and myoferlin can interact. We further verified this interaction using U2OS cells which express myoferlin but not dysferlin (Figure 5b). We performed IP experiments on non-transfected U2OS cells, and U2OS cells transiently transfected with recombinant dysferlin and analyzed IP fractions on western blot. As expected, F4 and H7 cannot immunoprecipitate myoferlin from wild-type U2OS cells (Figure 5b). We could only detect myoferlin, but not dysferlin, in the non-bound fractions, showing that U2OS cells only express myoferlin. This is also additional support that the HCAb are specific for dysferlin and cannot directly bind to myoferlin. However, myoferlin is co-immunoprecipitated from U2OS cells expressing recombinant dysferlin (Figure 5b, F4). Thus, F4 can immunoprecipitate myoferlin, albeit weakly, only when dysferlin is co-expressed. According to previous studies myoferlin is strongly expressed in myoblasts and prominently in mature muscle myonuclear membranes, and to a lesser extent at the sarcolemma.[15] Dysferlin is mostly observed at the sarcolemma. This indicates that only a small fraction of both proteins co-occur in muscle tissue, consistent with the observation that only a fraction of myoferlin co-immunoprecipitates with dysferlin. All together this indicates that myoferlin and dysferlin can be part of the same protein complex, and opens the possibility of their co-existence in a single protein complex in muscle.

### Bioinformatics analysis of all identified complex partners

We continued with the three data sets of putative dysferlin interacting proteins and performed bioinformatics analyses to characterize the dysferlin protein complex. We first analyzed the datasets with the STRING interaction database (http://string-db.org/). STRING contains information of known annotated protein-protein interactions. 14 dysferlin protein protein interactions are recorded in STRING for human and mouse, of which 6 are direct interactions described in PubMed references (CAV3, CAPN3, AHNAK, PARVB, ANXA1, ANXA2). The others are interactions inferred from co-occurrence in MedLine abstracts and protein databases. Protein-protein interactions described in recently published studies, such as TUBA [41], are not yet annotated, and therefore not included in STRING. The remaining 8 predicted dysferlin interactions in STRING are not supported by experimental evidence and include DGC partners. These interactions are not identified in our data sets, and we consider them unlikely given the existing literature on dysferlin. Thus, STRING does not contain any information on the dysferlin protein complex partners, and therefore we consider the majority of interactions identified with our IP experiments as novel dysferlin interactions.

### Literature analysis of the dysferlin protein complex

We then verified whether there was any prior support in the biomedical literature for the identified interactions of dysferlin by calculating and comparing the textual overlap in MedLine abstracts for dysferlin and other proteins (Figure S3). We used the concept profile text-mining technique.[44] In this method all concepts (e.g. proteins, diseases, chemicals, GO terms) that are associated with a given protein identifier in MedLine abstracts are assembled into a concept profile. The amount of overlapping concepts between two proteins is a measure of relatedness; it records both explicit (co-mentioning of two proteins in the same abstract) and implicit (both proteins co-mentioned with a third concept) relationships. We ranked all putative interaction partners based on their match scores and subsequently analyzed, through Area under the ROC Curve (AuC) analysis, whether the identified interaction partners of dysferlin were among the top ranked proteins. An AuC of 0.5 would imply that the identified proteins are ranked randomly over the whole range of proteins, while an AuC of 1 would imply that all top-ranked proteins co-immunoprecipiate with dysferlin. We obtained an AuC of ∼0.8 (Figure S3A), indicating that the proteins indentified in the IP experiments are related to dysferlin in MedLine abstracts, and may share similar functions and/or pathways. In Table 1A we list the top 9 proteins that share strongest conceptual overlap with dysferlin. This includes two of the described interaction partners (ANXA2 and TRIM72), the others are novel binding partners. To gain a better idea of which type of concepts associate with the dysferlin protein complex we clustered the identified proteins and subsequently annotated with concepts. Representative concepts for the observed clusters are shown in Table 1B (full table in S3), and include vesicle related concepts such as Kinesin Activity and Membrane Protein Traffic, consistent with the vesicular localization of dysferlin. However, also many novel associations are uncovered, such as Translation Initiation, Actin Cortical Patch, and Chaperonin Activity. These concepts may refer to novel dysferlin functionalities.

**Table 1:** Concept analysis of the identified dysferlin protein complex partners.

**A**	**Myoblasts**	**Myotubes**	**Tissue**
1	MYOF	MYOF	MYOF
2	VCL	TRIM72	VCL
3	EIF3B	LDB3	EIF3B
4	CAPZB	VCL	DCTN1
5	DCTN1	EIF3B	LMNA
6	ANXA2	CAPZB	HSP90B1
7	UTRN	ACTN2	NEDD4L
8	EIF2S1	TTN	HSPA5
9	LMNA	ANXA2	STAT1
**B**	**Myoblasts**	**Myotubes**	**Tissue**
1	kinesin activity	Translation Initiation	kinesin activity
2	dynein activity	protein disulfide isomerase activity	dynein activity
3	Myosin ATPase	chaperonin activity	Translation Initiation
4	membrane protein traffic	heat-shock response	chaperonin activity
5	Antigen Processing	ribonucleoprotein complex location	heat-shock response
6	Translation Initiation	ubiquitin activity	ubiquitin activity
7	transfer RNA	Actin myofilament	cell-cell adhesion
8	heat-shock response	phosphatase	citric acid cycle
9	chaperonin activity		Keratins
10	ribosomal protein activity		pre-replicative complex
11	ribonucleoprotein complex location		
12	Growth Cones		
13	actin cap		
14	actin cortical patch		
15	Cytoskeleton		
16	Intermediate Filaments		
17	ubiquitin activity		
18	nucleocytoplasmic transport		
19	phosphatase		
20	phosphatase		
21	cyclin-dependent protein kinase activity		

A) For each data set the proteins were assigned to concept profiles (summary of all concepts such as proteins, diseases and GO terms to associate with a given protein identifier), and conceptual overlap with dysferlin was calculated. The 9 proteins with strongest conceptual overlap are listed and include described interaction partners (in bold). B) Identified proteins were first clustered and subsequently annotated with concepts. Representative concepts for the uncovered clusters are shown for each data set (full table in S3).

Given that dysferlin expression is not restricted to skeletal muscle, we next evaluated co-expression of the dysferlin protein complex partners over multiple tissues, using the database Gene Atlas. We obtained an AuC of ∼0.7 (Figure S3B), where an AuC of 0.5 would mean a random level co-expression and an AuC of 1.0 would mean complete co-expression. This result suggests that we do not mainly identify muscle specific proteins.

### Gene Ontology analysis of the dysferlin protein complex

Gene Ontology (GO) category analysis is commonly used for bioinformatics analysis of large datasets. We used the DAVID web tool to analyze GO category enrichment by performing a cluster analysis (Table 2, full table in S4). Protein lists were uploaded in DAVID and compared to a random background data set (shown) or a random set of muscle genes (not shown) with comparable results. The output is a ranked set of GO term clusters that are significantly overrepresented in the IP data sets (Table 2). Many of the highest ranked clusters relate to vesicle trafficking (in bold). Examples of such proteins are Clathrin and Rab GTPases. This corroborates the reported function of dysferlin as a vesicular protein. In addition to vesicle trafficking, several other clusters are significantly represented, which may point to potential novel roles for dysferlin function.

**Table 2:** DAVID annotation of clusters in the IP lists.

	Myoblasts	Myotubes	Tissue	Core set
1	GO:0006412∼translation	GO:0043292∼contractile fiber	GO:0005524∼ATP binding	GO:0006457∼protein folding
2	GO:0005524∼ATP binding	GO:0043232∼intracellular non-membrane-bounded organelle	GO:0006457∼protein folding	GO:0005524∼ATP binding
3	GO:0043232∼intracellular non-membrane-bounded organelle	GO:0005524∼ATP binding	GO:0043232∼intracellular non-membrane-bounded organelle	GO:0043232∼intracellular non-membrane-bounded organelle
4	**GO:0016023∼cytoplasmic membrane-bounded vesicle**	GO:0006412∼translation	GO:0043623∼cellular protein complex assembly	**GO:0016023∼cytoplasmic membrane-bounded vesicle**
5	**GO:0015031∼protein transport**	**GO:0016023∼cytoplasmic membrane-bounded vesicle**	GO:0006412∼translation	GO:0043292∼contractile fiber
6	GO:0006457∼protein folding	GO:0006457∼protein folding	**GO:0007018∼microtubule-based movement**	GO:0006986∼response to unfolded protein
7	GO:0006413∼translational initiation	GO:0006096∼glycolysis	**GO:0016023∼cytoplasmic membrane-bounded vesicle**	GO:0042623∼ATPase activity, coupled
8	GO:0005739∼mitochondrion	GO:0015629∼actin cytoskeleton	GO:0006986∼response to unfolded protein	**GO:0007018∼microtubule-based movement**
9	GO:0008180∼signalosome	GO:0005739∼mitochondrion	GO:0030529∼ribonucleoprotein complex	**GO:0031974∼membrane-enclosed lumen**
10	**GO:0003924∼GTPase activity**	GO:0045333∼cellular respiration	***GO:0015031∼protein transport***	**GO:0006412∼translation**
11	GO:0030036∼actin cytoskeleton organization	GO:0005882∼intermediate filament	GO:0005882∼intermediate filament	GO:0030017∼sarcomere
12	GO:0006418∼tRNA aminoacylation for protein translation	GO:0000502∼proteasome complex	**GO:0031974∼membrane-enclosed lumen**	GO:0000502∼proteasome complex
13	GO:0006096∼glycolysis	GO:0006418∼tRNA aminoacylation for protein translation	GO:0006163∼purine nucleotide metabolic process	GO:0005882∼intermediate filament
14	GO:0005882∼intermediate filament	GO:0006936∼muscle contraction	GO:0016887∼ATPase activity	GO:0006418∼tRNA aminoacylation for protein translation

All three datasets were analyzed separately. Protein lists were uploaded in DAVID and analyzed against a random set of genes as background. A p-value was calculated for all GO terms, and those GO terms that were significantly overrepresented compared to the background set were clustered. Subsequent ranking of clusters is determined by the combined p-value. For each cluster a representative GO term is shown in the table. Full tables are in S4. The pathways that relate to vesicles are enhanced in bold.

Many identified proteins relate to ATP binding and purine synthesis. This finding is interesting as a recent study showed that dysferlin is involved in extracellular ATP and ADP signaling. One of the indentified target proteins, found in all data sets, is ATP synthase (ATP5b).

A large number of mitochondrial proteins is indentified in the dysferlin protein complex. 18 proteins are observed in the core-set of 115 proteins. This includes proteins that localize to the outer and inner mitochondrial membrane, indicating that large parts of mitochondrial membranes co-purify with dysferlin. Intriguingly, the alternative first exon of dysferlin [45] encodes a putative mitochondrial targeting signal (not shown), raising the possibility that dysferlin is targeted to mitochondria in myoblasts.

Finally, among the top ranked clusters in all three data-sets, including the core set of 115 proteins, are clusters that relate to protein translation and folding. A possible explanation for this is that ER-residing dysferlin is immunoprecipitated from the cells.

### KEGG pathway analysis of the dysferlin protein complex

We proceeded with a KEGG pathway analysis, which gives a better visualization how the identified dysferlin protein complex partners relate to each other (table S5). Interestingly, the strongest vesicle-related KEGG pathway in all three data sets is endocytosis, rather than exocytosis. This is consistent with previous studies which suggested that dysferlin undergoes rapid endocytosis in the absence of caveolin 3 on the cell membrane.[19] Additionally, dysferlin negative cells show an upregulation of endocytotic proteins [46], again suggesting a role for dysferlin in endocytosis. Most proteins that are found upregulated by Nagaraju *et al* [46] are coimmunoprecipitated with dysferlin, and include Mannose-6 phosphate receptor (CIMPR, or IGF2R), adaptin (AP2), clathrin-α (CLTA) and the GTPase RAC2 (Table S1). Interestingly, this KEGG pathway analysis also revealed several immunological pathways, such as Fc gamma R-mediated phagocytosis. As dysferlin is also expressed in monocytes [47]–[49], this indicates that common pathways may exist between muscle and monocytes.

Multiple pathways are ranked higher than Endocytosis, but do not relate directly to vesicles. The top-ranked pathway is Metabolic Enzymes, which includes most of the mitochondrial proteins identified in the IPs. This indicates a role for dysferlin in energy metabolism. However, due to the high content of metabolic enzymes in muscle, some aspecific interactions cannot be excluded. Of interest is the presence of creatine kinase M and B. Serum levels of creatine kinase are exceptionally high in dysferlinopathy patients, reaching 100 fold above normal.[50]

Finally, the pathways Focal Adhesion and Tight Junction are ranked high in all 3 data sets. Focal adhesions and tight junctions are two structures involved in cell-cell and cell-matrix contacts, in which the intracellular actin cytoskeleton is linked by transmembrane proteins to extracellular proteins. These adhesion sites exist independently of the DGC, and previous cellular fractionation experiments have shown that integrins, which are part of focal adhesions, are restricted to different membrane sections than the DGC.[51] While we do not identify DGC components in the dysferlin protein complex, several focal adhesion proteins were identified in all three sources, including vinculin (VINC), actinin (ACTN), and talin (TLN). This hints at a role for dysferlin in cell adhesion.

In all, the three datasets indicate dysferlin function beyond muscle membrane repair.

### Dysferlin can interact with the focal adhesion component vinculin

**Figure 6:**
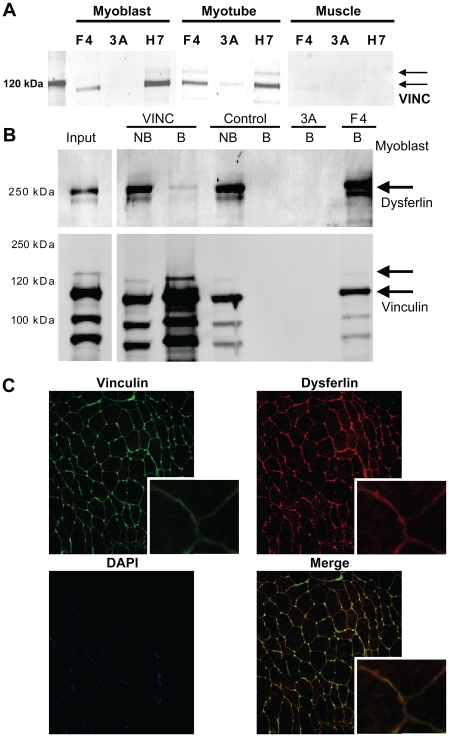
A potential interaction between dysferlin and vinculin. A) The dysferlin IP samples were analyzed on western blot for vinculin, to verify that full-length vinculin specifically co-immunoprecipitates with dysferlin, but not with the 3A control HCAb. B) cell lysate of IM2 myotubes were subjected to immunoprecipitate with antibodies against vinculin and an IgG control (against VSV), and IP and non-bound fractions were analyzed for dysferlin on western blot. HCAb F4 and 3A were taken along as positive and negative controls, respectively. Dysferlin is strongly detected in the F4 fraction (upper panel). Weak signal is also seen in the vinculin bound fractions, indicating a weak interaction with dysferlin. A corresponding western blot for vinculin shows the presence of full-length vinculin in the F4 and vinculin IP fractions (lower panel). C) Human skeletal muscle cryosections were stained for vinculin (green) and dysferlin (F4, red). In cross-sections (right) both dysferlin and vinculin are detected at the sarcolemma.

To validate our bioinformatics analysis we further investigated one of the newly identified pathways: Focal Adhesion. Vinculin has strong conceptual overlap with dysferlin (VCL in Table 1A). Interestingly, the previously reported dysferlin interactor PARVB localizes to and regulates focal adhesions.[42] PARVB can directly interact with ILK and dysferlin, and regulates focal adhesion turnover.[42], [52], [53] In the absence of dysferlin, PARVB is no longer recruited to the sarcolemma.[42] To confirm the existence of focal adhesion components in the dysferlin protein complex we verified that both dysferlin HCAbs can co-immunoprecipitate full-length vinculin (VINC) (Figure 6a). A vinculin doublet, corresponding to two vinculin isoforms [54], is detected consistently in the dysferlin HCAb IP fractions, but not in controls. Interestingly, the relative amount of immunoprecipitated vinculin decreases with differentiation, suggesting that the interaction is dynamic. To further support the validity of this interaction we performed the reverse experiment and used conventional antibodies against vinculin to co-immunoprecipitate dysferlin (Figure 6b). These antibodies can efficiently co-immunoprecipitate dysferlin from cultured myotubes, unlike isotype control antibodies, indicating the validity of their interaction. We performed additional immunofluorescent analysis of human skeletal muscle cryosections. As shown in Figure 6c, vinculin is found predominantly at the sarcolemma in cross-sections, and colocalizes with dysferlin.

## Discussion

We have identified a large set of potential dysferlin protein complex partners in a myogenic context. We showed that many new complex partners participate in vesicle trafficking, which corroborates previous data that dysferlin is involved in this process and suggest that its interaction partners, such as Clathrin, assist in this function. In addition we identified several potential novel roles for dysferlin, including cell adhesion, metabolism, mitochondrial association, and immune cell function.

F4 and H7 show a different performance in the IP as shown in Figure 2. Such differences in performance are not unique to HCAb, and it is difficult to predict and establish why this occurs. It might be that the antibodies compete with interaction partners at the site of antigen binding, or that the epitope availability is not uniform. The latter has been described for dysferlin as C2 domains are calcium sensitive [7], and VHH are known to recognize three dimensional folds [55].

It must be noted that the IP procedure does not discriminate between direct and indirect interactions. We aimed to limit the potential problem of non-specific binders by analyzing two dysferlin specific HCAb and using different myogenic sources and buffers with different solubilizing properties. Although it cannot be excluded that some co-immunoprecipitated proteins are non-specific binders, the large overlap of immunoprecipiated proteins in all three sources under different stringency conditions, and their absence in the control experiments, indicates that most IP proteins are valid complex partners of dysferlin, whether direct or indirect. In addition, we tested and confirmed 8 of 9 established protein-protein interactions of dysferlin, and provided biochemical evidence for the novel interactions with myoferlin and vinculin. Finally, an additional indication for the validity of the method is that no components of the DGC were identified.

Myoferlin was consistently identified in the dysferlin protein complex. Given the specificity of all antibodies used in this study we do consider this interaction to be genuine. It is interesting to speculate that dysferlin could form oligomers with its homologous family member myoferlin, and how this contributes to protein function, possibly patch-fusion repair.

115 Proteins were consistently immunoprecipitated from all three protein sources. These proteins we consider to be the strongest candidates for dysferlin complex partners, or represent functionalities of dysferlin that do not change with differentiation. The proteins that were identified only in a subset of sources can be explained in three ways: 1) by expression differences (eg. MG53 (TRIM72) is not expressed in myoblasts and therefore only immunoprecipitated from myotubes, Table S1), 2) as non-specific binders, or 3) representing a change in dysferlin function during muscle differentiation.

We often observed in our western blots that the relative amounts of immunoprecipitated proteins often changes with differentiation. Examples of such dynamic protein-protein interactions of dysferlin are calpain 3 (Figure 2B, C) myoferlin (Figure 5A) and vinculin (Figure 6a). This suggests that the dysferlin protein complex has a dynamic nature as a function of myogenic differentiation. When we further compared the specific proteins for each dataset, we noticed that a high number of mitochondrial proteins was co-immunoprecipitated from myoblasts (20%, not shown). This amount decreases with differentiation and is lowest in tissue (12%). In contrast, the relative amount of metabolic enzymes from the glycolysis pathway is enriched with differentiation (going from 2% to 4%). This may indicate that the function of dysferlin changes with differentiation. Unfortunately our qualitative analysis does not allow for solid quantitative conclusions due to its dependence on experimental conditions. Future studies using quantitative proteomic approaches may give a clearer impression of the relative abundance of the protein identified in the dysferlin protein complex during certain processes.

Among the identified mitochondrial proteins is ATP synthase (ATP5b). ATP synthase is a protein complex enriched in mitochondria and part of the respiratory chain.[56] In addition ATP5b is also found at the cell membrane, where it maintains extracellular ATP and ADP levels, and functions as a membrane receptor for HDL.[56] It was recently shown that dysferlin is involved in extracellular ATP and ADP signaling.[34] It is tempting to speculate that the putative interaction between Dysferlin and ATP synthase allows for the formation of vesicle with ATP content to participate in the repair process.

Many of the identified novel dysferlin protein interactions relate to protein translation and folding. It was recently suggested that pathogenic mutations in dysferlin result in improper folding of the protein and therefore in dysfunction and degradation.[57] Moreover, it has been shown that the ER can function as an intracellular storage compartment of recombinant dysferlin [58], which is in line with elongated translation/folding time. Therefore we feel that the identified interactions between dysferlin and several ER proteins are not an artifact but reflect the amount of effort that the muscle cells invest into proper folding of the 230 kDa protein.

It has been shown that most of recombinant dysferlin localizes to the ER, and that ER-residing recombinant dysferlin can be targeted to two distinct routes of degradation: ERAD and stress-induced autophagy.[58] The latter process depends on phosphorylation of EIF2 and the autophagy gene MAP1/LC3.[58] Interestingly, both proteins were identified to co-immunoprecipitate with endogenous dysferlin (Table S1), suggesting that a similar autophagy route also exists for endogenous dysferlin in vivo. In addition, it was shown that recombinant dysferlin interacts with the chaperones VCP and SEC61 [58] as part of the ERAD pathway. Both VCP and SEC61 co-immunoprecipitate with endogenous dysferlin.

Based on the high ranking of the Focal Adhesion KEGG pathway in all data sets we focused our attention on this pathway. Mutations in the focal adhesion components ITGA7[59], and VINC[54] result in a muscle pathology. Interestingly, integrins and FAK have been shown to be essential for myogenic differentiation and localize at the site of fusion.[60]–[62] Dysferlin shows a similar localization in differentiated myotubes.[33] This indicates that dysferlin might be involved in regulation of cell-cell contacts. Interestingly, a recent screen identified integrins (the transmembrane components of focal adhesions) to be essential for endocytosis in HeLa cells.[63] This is in line with our finding that dysferlin co-immunoprecipitates endocytic proteins.

Dysferlin was previously described to be involved in membrane repair. For membrane repair to occur, the cell needs to patch the damaged membrane, and remodel the local cytoskeleton [26]. It has been shown that at the site of damage, cytoskeletal proteins talin and vimentin are proteolytically processed by calpains.[26] Moreover, at this site integrins and actin rapidly accumulate [26], suggesting cytoskeletal remodeling of the cortical cytoskeleton, and the adhesion sites. It is tempting to speculate that dysferlin is involved in both processes. Through its membrane association it can target vesicular membrane stores, and through its interaction with focal adhesion components it can coordinate cytoskeletal remodeling. Previous work showed that dysferlin recruits PARVB to the sarcolemma.[42] PARVB can directly interact with integrin linked kinase, and is important for stabilizing focal adhesions.[52], [53] In the absence of dysferlin PARVB does not localize to the sarcolemma, but the functional consequence of this is unknown.[42] Calpain 3 is also in complex with dysferlin. Through its proteolytic activity it can modulate the direct interaction between dysferlin and the giant protein AHNAK.[24] Thus, dysferlin might act as a sensor to coordinate the remodeling of structural proteins in addition to aiding patch-fusion of membranes.

In conclusion we observed that dysferlin participates in diverse processes in a spatiotemporal dependent manner, that together ensure proper maintenance of skeletal muscle membrane integrity.

## Methods

### Cell culture

Mouse IM2 myoblast were maintained under permissive conditions at 33°C and 10% CO2 in DMEM (61965, GIBCO) supplemented with L-Glutamine, 1% Pen/Strep (100IU/100UG/ML, Gibco-BRL), 20% FCS, IFN-γ, and Chick embryo extract. For differentiation cells were grownt to 70% confluency, washed with PBS and grown in DMEM (61965) supplemented with 10% HS, L-Glutamine, and Pen/Strep.

### Antibodies

The following antibodies were used in this study: MaCAPN3 (NCL-12A2 Novocastra) at 1;500 for western blot. MaVSV (P5D4 gift from Dr. J. Fransen, Nijmegen, The Netherlands) at 1;5,000 for IF. MaDYSF (Hamlet, Novocastra) at 1;300 for western blot and IHC. MaMYOF (gift from Dr. R. Bashir), GaMousealexa488 (Molecular Probes, Eugene, OR, USA), GaRabbitalexa594 (Molecular Probes) at 1;1,000 and 1;2,000, respectively. GaRabbitIRDye800 and GaMouseIRDye680 (Westburg) were used at 1;5,000 for western blotting.

### Immunoprecipitation

The following lysis buffers were used: Triton buffer (50 mM TrisHCl, pH 7.5, 150 mM NaCl, 0.2% Triton X100, 1x protease inhibitor cocktail (Roche)), CHAPS buffer (50 mM Tris-HCl pH 7.5, 150 mM NaCl, 0.15% CHAPS and 1x protease inhibitor cocktail), or SDS buffer (20 mM triethanolamine 0,14 M NaCl, 0,1% DOC, 0,1% SDS, 0,1% triton X100, protease inhibitor cocktail). Cultured cells were prepared freshly by washing in PBS and lysed by scraping on ice in cold lysis buffer. Proteins Snap-frozen human skeletal muscle tissue was homogenized in CHAPS buffer. All homogenates were spun down at maximum speed, 4°C, 20 min. The pellet was discarded as debris. The supernatant containing the solubilized proteins was cleared with protein A Sepahrose CL-4B (Amersham). The Sepharose was first pre-equilibrated by a 3x wash in lysis buffer and added to the homogenates for 1 h, at 4°C tumbling. Sepharose was removed and antibody added (50 µg HCAb, or 10 µg conventional antibody) for O/N incubation at 4°C tumbling. Thereafter pre-equilibrated Sepharose was added and incubated for 2 h, 4°C, tumbling). Homogenates were spun down at 500 g and supernatant stored as non-bound fraction. The Sepharose was washed 5x (>5 min tumbling at 4°C). Finally, all fluid was removed and protein eluted by boiling in sample buffer. This was analyzed as the IP or bound fraction.

### Western blot

Cell lysates were prepared in Llaemli sample buffer and loaded onto SDS-PAGE gels. Proteins were separated and blotted onto PVDF membranes. Blots were washed, blocked in 4% mPBS for 30 min, and incubated with primary antibody diluted in blocking buffer for 2 h at RT. Subsequent washes in PBS-tween were followed with 1 h incubation with secondary antibody in the dark. Blots were washed and scanned with an odyssey scanner (Licor, Lincoln, Nebraska, USA).

### Immunostaining

For immunohistochemical examinations, muscle cryosections of 6 µm thickness were fixed in 3.7% formaldehyde in phosphate-buffer saline (PBS) containing 0.1% Triton X-100 for 30 min, following by pre-incubation with 4% skimmed milk (Marvel) in PBS at room temperature for 2 h. The sections were incubated with primary antibody o/n at 4°C, and subsequently by incubation of fluorescein-labeled secondary antibody for 1 h min at RT. The sections were washed with PBS, dehydrated with (subsequently) 70, 90, 100% ethanol and mounted in a DAPI (50 ng/µl)/ Vectashield mounting medium (Burlingame). Final preparations were analyzed with a Leica Aristoplan fluorescence microscope and images were obtained using a Cytovision (Applied imaging) digital system.

### In-gel tryptic digestion

IP fractions were separated on SDS-PAGE gels, and proteins were visualized with Coomassie (SimplyBlue, Invitrogen). Gel lanes were sliced into 25–30 bands, cut into small pieces and washed with 25 mM NH4HCO3 followed by two rounds of dehydration with 100% acetonitrile for 10 min. For reduction and alkylation, gel particles were first incubated with 10 mM dithiothreitol for 30 minutes at 56 °C. Following dehydration with acetonitrile, gel plugs were subsequently incubated in 55 mM iodoacetamide for 20 minutes at room temperature. After two rounds of washing with 25 mM NH4HCO3 and dehydration with 100% acetonitrile, the gel particles were completely dried in a centrifugal vacuum concentrator (Eppendorf, Hamburg, Germany). Dried gel particles were re-swollen for 15 min. on ice by addition of 15 µl of a trypsin solution (12.5 ng/µl in 25 mM NH4HCO3, Sequencing grade modified trypsin, Promega, Madison, WI). Following this, 20 µl of 25 mM NH4HCO3 was added and samples were kept on ice for an additional 30 min. Tryptic digestion was subsequently performed overnight at 37 °C. Following tryptic digestion, the overlaying digestion-solution was collected. Two additional rounds of extraction with 20 µl 0.1% TFA were used to extract peptides from the gel plugs and all extracts were pooled.

### Nano LC ESI MS/MS

Nanoflow LC was performed on an Ultimate LC system (Dionex, Sunnyvale, CA). A volume of 10 µL of sample was injected onto a C18 PepMapTM 0.3 mm×5 mm trapping column (Dionex) and washed with 100% A (2% acetonitrile in 0.1% formic acid in MQ water, v/v) at 20 µL/min for 15 min. Following valve switching, peptides were separated on a C18 PepMap 75 µm×150 mm column (Dionex) at a constant flow of 200 nL/min. The peptide elution gradient was from 10 to 60% B (95% acetonitrile in 0.1% formic acid in MQ water v/v) over 50 min. The nanoflow LC system was coupled to an HCTultra IonTrap (Bruker Daltonics, Bremen, Germany) using a nano-electrospray ionisation source. The spray voltage was set at 1.2 kV and the temperature of the heated capillary was set to 165 °C. Eluting peptides were analyzed using the data dependent MS/MS mode over a 300–1500 m/z range. The five most abundant ions in an MS spectrum were selected for MS/MS analysis by collision-induced dissociation using helium as the collision gas.

### Mass spectrometry data analysis

Peak lists were generated using DataAnalysis 4.0 software (Bruker Daltonics) and exported as Mascot Generic (MGF) files. These files were searched against the mouse (myoblast and myotube samples) or the human (muscle sample) IPI database using the Mascot (version 2.2.1) search algorithm (Matrix Science, London, UK) and data from 1 lane were merged using Mascot Deamon. An MS tolerance of 0.6 Da (with # 13C = 1) and a MS/MS tolerance of 0.5 Da was used. Trypsin was designated as the enzyme and up to one missed cleavage site was allowed. Carbamidomethylcysteine was selected as a fixed modification and oxidation of methionine as a variable modification. Only significant protein hits with at least one unique peptide with a score above 30 were selected. All identified proteins were assigned to concepts. Proteins for which insufficient data was available to create a concept were discarded. To make sure that the remaining “single peptide” hits did not strongly influence the data analysis we performed the bioinformatics analyses of the myoblast data with a more stringent data set requiring a minimum of 2 unique peptides, and obtained comparable results (not shown).

### Text-mining analysis

To calculate the similarity of the contexts in which proteins appear in literature, we summarize the context of each protein in a concept profile. This profile contains all concepts that have a direct relation with a protein as found in Medline abstracts. The concepts in a profile include, in addition to proteins, all other concepts described in the Unified Medical Language System (UMLS), such as diseases, symptoms, tissues, biological processes and many other types of concepts. For a detailed description of concept profiles we refer to Jelier *et al*.[44] The similarity score between two concept profiles is taken as the inner product of the concept profile vectors.

### Co-expression analysis

Microarray co-expression data (human GNF1H chip) were downloaded from Gene Atlas (http://biogps.gnf.org/downloads/). First the log was taken from the MAS5.0 normalized expression values for each tissue (78 in total). Then a Pearson correlation was calculated over these values.

### Bioinformatics

IPI numbers retrieved from Mascot were mapped to GENE identifiers using the NCBI mapping. GENE lists were analyzed using the webtool DAVID (http://david.abcc.ncifcrf.gov/), using functional annotation clustering. The following settings were used: GOTERM_BP_FAT, GOTERM_CC_FAT, GOTERM_MF_FAT, COG_ONTOLOGY, SP_PIR_KEYWORDS, UP_SEQ_FEATURE. KEGG Pathways were analyzed separately. The analyses were done with a random gene set as background, and subsequently with a muscle specific (obtained from C2C12 micro-array experiments) gene set, for confirmation.

A Functional Annotation Clustering was performed, and rank determined by the software, based on an enrichment score calculated from the separate p-values for each GO-term associated with a cluster. Whole output tables are included in the supplement. Representative GO terms for each cluster are included in summarizing Table 1.

Separately, the gene lists were analyzed with the web tool KEGG (http://www.genome.jp/kegg/), using object search in KEGG pathways. Random gene lists were used as reference.

## Supporting Information

Figure S1 Western blot of Dysferlin IP. Dysferlin was immunoprecipitated from IM2 myoblasts, IM2 myotubes and human skeletal muscle tissue. Input, non-bound (NB) second wash, and bound (B) fractions were analyzed on western blot for Dysferlin and Tubulin content. Arrows denote the protein bands. As expected Dysferlin is detected in all input and non-bound fractions. In addition it is also identified in the bound fractions for F4 and H7, but not the negative control IP 3A. Tubulin has a similar pattern.
(0.57 MB TIF)

Figure S2 Western blot for reported dysferlin interaction partners. Dysferlin IP samples were probed on western blot for Dysferlin, AHNAK, and Calpain 3. AHNAK and Calpain 3 specifically co-immunoprecipitate with Dysferlin. Arrows denote the protein bands.
(0.57 MB TIF)

Figure S3 Concept profiling and co-expression analysis. The area under the ROC curve (AuC) was calculated for concept profiles, and is plotted for all thee datasets. The AuC is 0.76 for Proliferation, 0.78 for Differentiation, 0.77 for Tissue. B) The AuC was calculated for GeneAtlas, and is plotted for all thee datasets. The AuC is 0.72 for Proliferation, 0.73 for Differentation, 0.73 for Tissue.
(0.11 MB TIF)

Table S1 Proteins identified in the dysferlin protein complex. For each protein the IPI reference, Official Gene Symbol, and EntrezGene Identifier are shown.
(0.11 MB XLS)

Table S2 List of proteins identified by mass spectrometry. The number of total and unique peptides is limited to peptides with an individual Mascot score above 30 and the protein coverage is based on these peptides only. The tables are ranked based on emPAI scores [64].
(0.20 MB XLS)

Table S3 Conceptual analysis of the dysferlin protein complex. Identified proteins were clustered in the webtool Anni, and the clusters subsequently annotated with concepts. For each cluster the associated proteins are shown, together with a representative associated concept.
(0.03 MB XLS)

Table S4 DAVID analysis of the dysferlin protein complex. Identified proteins were uploaded into DAVID and analyzed against a background set of random proteins. Protein were clustered based on GO terms, and strongest overrepresented clusters are shown. In bold is the representative GO term that is shown in Table 2.
(1.09 MB XLS)

Table S5 KEGG pathway representation. Pathways that relate to calcium signaling and vesicle trafficking are in bold, the other pathways indicate potential new roles of dysferlin. Of interest are the immunoregulatory processes that relate to antigen processing, phagocytosis and migration, as dysferlin is expressed in immune cells. For each pathway the number of associated genes is given. The disease-linked pathways Huntington, Parkinson and Alzheimer disease, refer to metabolic, mitochondrial enzymes, and reflect signaling pathways secondary to those diseases.
(0.05 MB DOC)
