# Supplementary figures and images for: Proteomic Analysis of the Dysferlin Protein Complex Unveils Its Importance for Sarcolemmal Maintenance and Integrity
Source: PLoS One. 2010 Nov 5;5(11):e13854. doi: 10.1371/journal.pone.0013854 (PMC2974636; doi:10.1371/journal.pone.0013854)

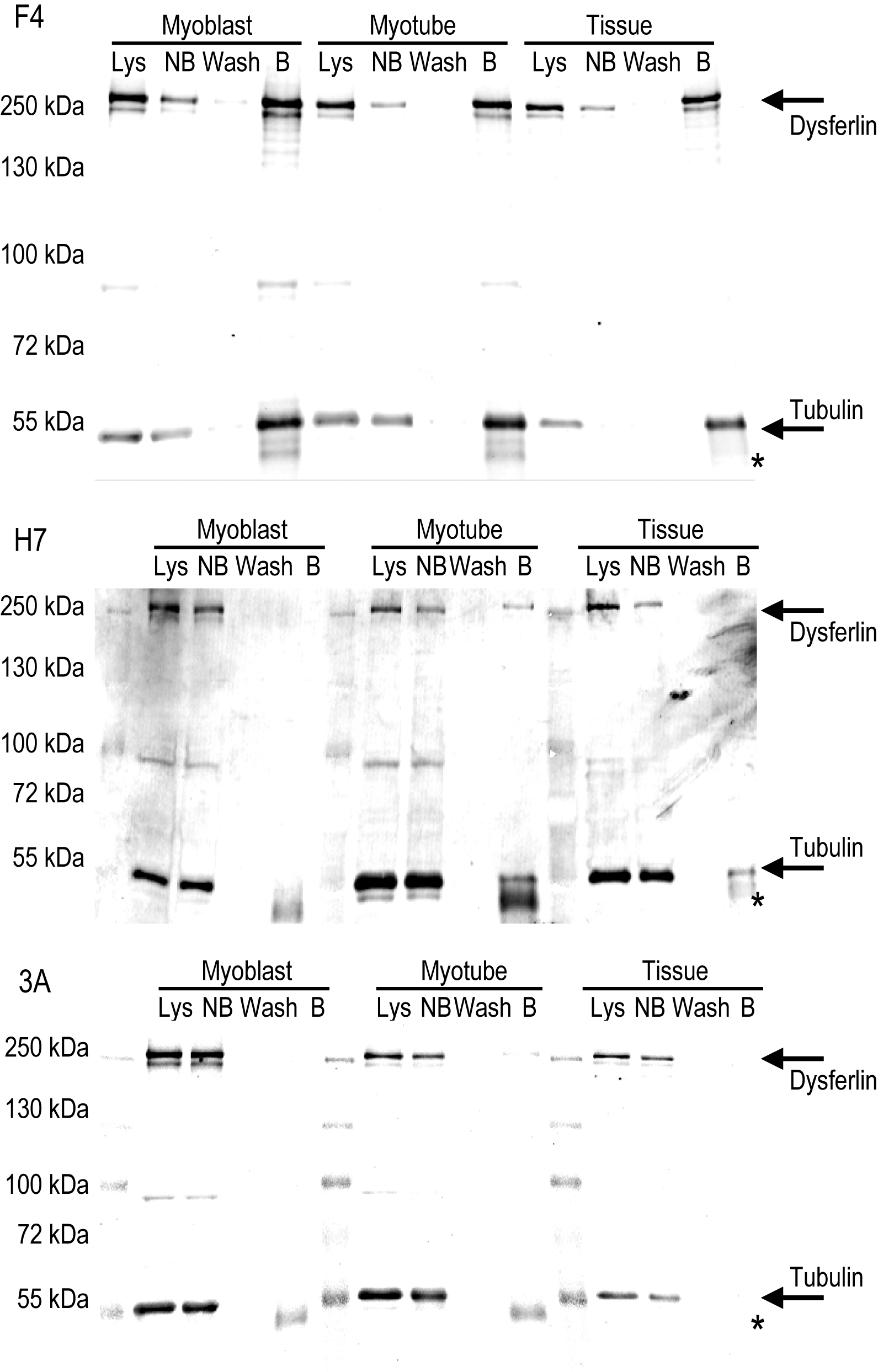

Supplement: Figure S1 — Western blot of Dysferlin IP. Dysferlin was immunoprecipitated from IM2 myoblasts, IM2 myotubes and human skeletal muscle tissue. Input, non-bound (NB) second wash, and bound (B) fractions were analyzed on western blot for Dysferlin and Tubulin content. Arrows denote the protein bands. As expected Dysferlin is detected in all input and non-bound fractions. In addition it is also identified in the bound fractions for F4 and H7, but not the negative control IP 3A. Tubulin has a similar pattern. (0.57 MB TIF) [file pone.0013854.s001.tif]

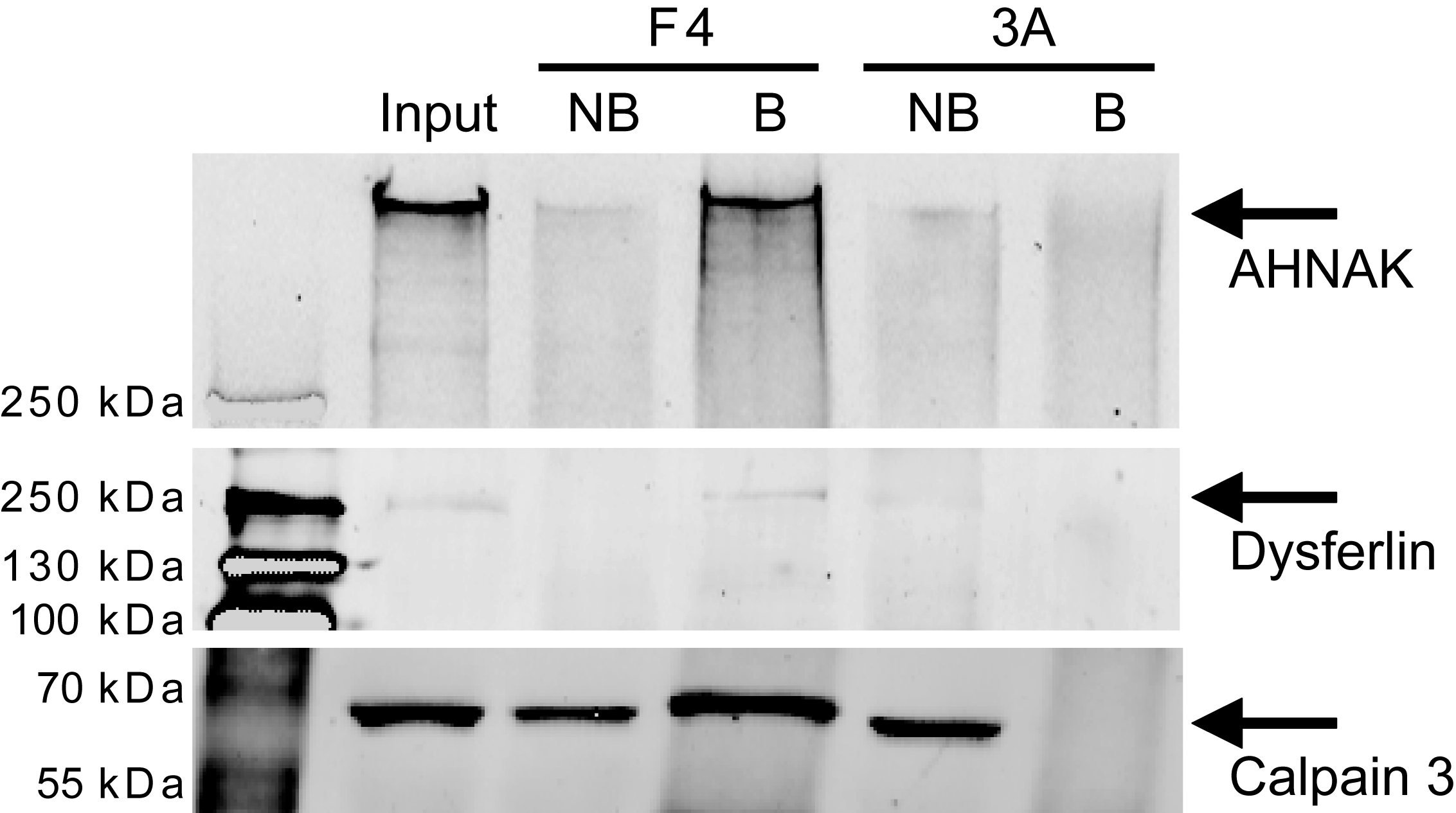

Supplement: Figure S2 — Western blot for reported dysferlin interaction partners. Dysferlin IP samples were probed on western blot for Dysferlin, AHNAK, and Calpain 3. AHNAK and Calpain 3 specifically co-immunoprecipitate with Dysferlin. Arrows denote the protein bands. (0.57 MB TIF) [file pone.0013854.s002.tif]

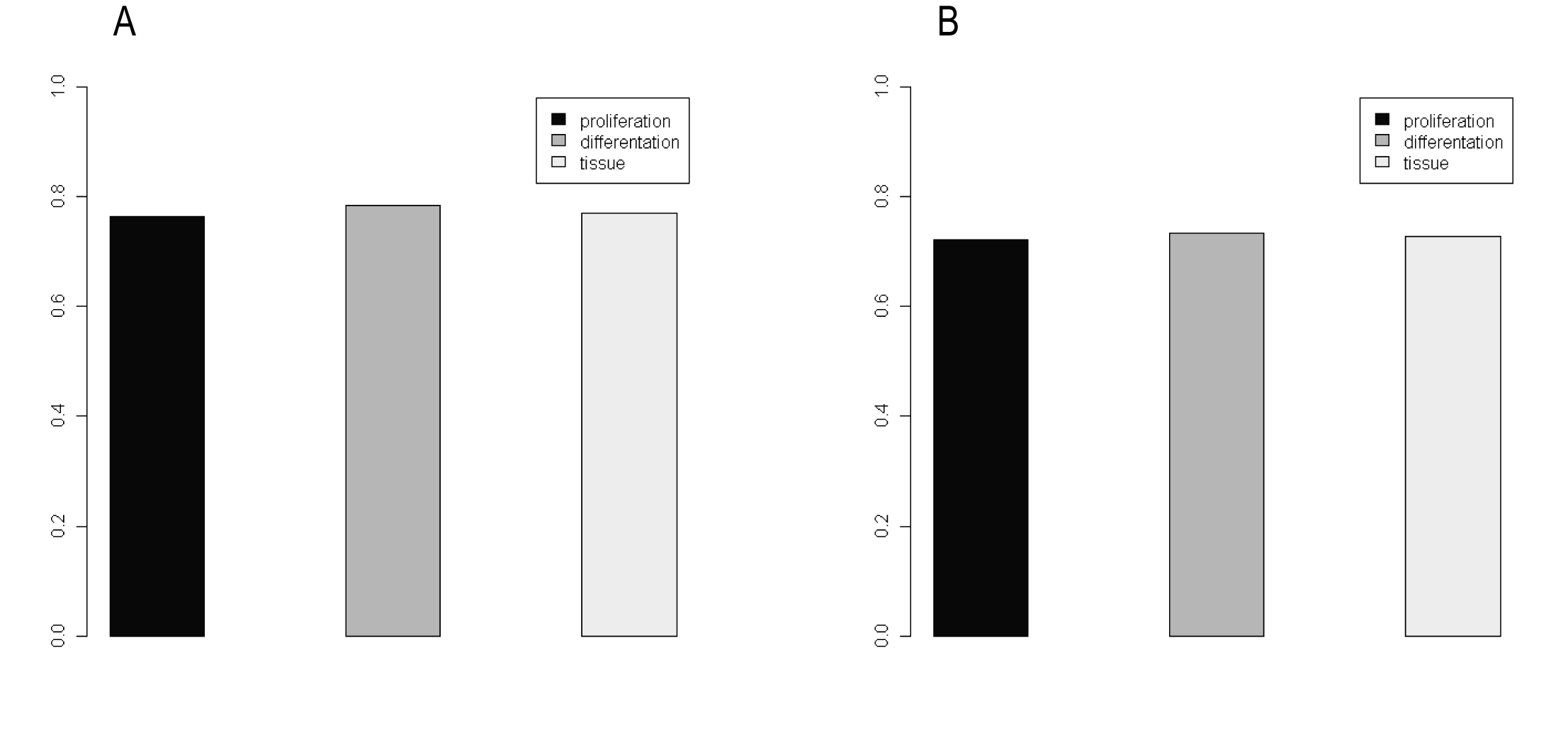

Supplement: Figure S3 — Concept profiling and co-expression analysis. The area under the ROC curve (AuC) was calculated for concept profiles, and is plotted for all thee datasets. The AuC is 0.76 for Proliferation, 0.78 for Differentiation, 0.77 for Tissue. B) The AuC was calculated for GeneAtlas, and is plotted for all thee datasets. The AuC is 0.72 for Proliferation, 0.73 for Differentation, 0.73 for Tissue. (0.11 MB TIF) [file pone.0013854.s003.tif]
