# Supplementary material for: Proteomic Analysis of the Dysferlin Protein Complex Unveils Its Importance for Sarcolemmal Maintenance and Integrity
Source: PLoS One. 2010 Nov 5;5(11):e13854. doi: 10.1371/journal.pone.0013854 (PMC2974636; doi:10.1371/journal.pone.0013854)
Supplement: Table S5 — KEGG pathway representation. Pathways that relate to calcium signaling and vesicle trafficking are in bold, the other pathways indicate potential new roles of dysferlin. Of interest are the immunoregulatory processes that relate to antigen processing, phagocytosis and migration, as dysferlin is expressed in immune cells. For each pathway the number of associated genes is given. The disease-linked pathways Huntington, Parkinson and Alzheimer disease, refer to metabolic, mitochondrial enzymes, and reflect signaling pathways secondary to those diseases. (0.05 MB DOC) [file pone.0013854.s008.doc]

|  | **Myoblasts** | **Myotubes** | **Tissue** | **Core set** |
| --- | --- | --- | --- | --- |
| 1 | # mmu01100 Metabolic pathways - (81) | # mmu01100 Metabolic pathways - (64) | # hsa01100 Metabolic pathways - (43) | mmu01100 Metabolic pathways (23) |
| 2 | # mmu05016 Huntington's disease - (25) | # mmu00020 Citrate cycle (TCA cycle) - (17) | # hsa05130 Pathogenic Escherichia coli infection - (12) | mmu04510 Focal adhesion (7) |
| 3 | # mmu05012 Parkinson's disease - (21) | # mmu05010 Alzheimer's disease - (15) | # hsa04530 Tight junction - (11) | mmu03050 Proteasome (6) |
| 4 | # mmu04510 Focal adhesion - (19) | # mmu05012 Parkinson's disease - (14) | # hsa04510 Focal adhesion - (11) | mmu00010 Glycolysis / Gluconeogenesis (6) |
| 5 | # mmu04530 Tight junction - (18) | # mmu05016 Huntington's disease - (14) | # hsa03040 Spliceosome - (11) | mmu04540 Gap junction (5) |
| 6 | # mmu05010 Alzheimer's disease - (18) | # mmu04510 Focal adhesion - (14) | # hsa04110 Cell cycle - (10) | mmu04530 Tight junction (5) |
| 7 | # mmu04810 Regulation of actin cytoskeleton - (18) | # mmu00190 Oxidative phosphorylation - (13) | # hsa00230 Purine metabolism - (10) | mmu05412 Arrhythmogenic right ventricular cardiomyopathy (5) |
| 8 | # mmu03040 Spliceosome - (18) | # mmu00010 Glycolysis / Gluconeogenesis - (13) | # hsa00010 Glycolysis / Gluconeogenesis - (9) | mmu05200 Pathways in cancer (5) |
| 9 | # mmu00190 Oxidative phosphorylation - (17) | # mmu04530 Tight junction - (12) | # hsa04540 Gap junction - (9) | mmu04520 Adherens junction (5) |
| 10 | # mmu03010 Ribosome - (16) | # mmu04810 Regulation of actin cytoskeleton - (12) | # hsa05016 Huntington's disease - (8) | mmu04670 Leukocyte transendothelial migration (5) |
| 11 | # mmu03050 Proteasome - (14) | # mmu03050 Proteasome - (12) | # hsa03050 Proteasome - (8) | mmu00020 Citrate cycle (TCA cycle) (5) |
| 12 | # mmu04110 Cell cycle - (14) | # mmu00970 Aminoacyl-tRNA biosynthesis -(11) | # hsa04520 Adherens junction - (8) | mmu05012 Parkinson's disease (4) |
| 13 | # mmu04114 Oocyte meiosis - (13) | # mmu04114 Oocyte meiosis - (10) | # hsa05412 Arrhythmogenic right ventricular cardiomyopathy - (7) | mmu04114 Oocyte meiosis (4) |
| 14 | # mmu00970 Aminoacyl-tRNA biosynthesis - (12) | # mmu03010 Ribosome - (10) | # hsa05200 Pathways in cancer - (7) | mmu04810 Regulation of actin cytoskeleton (4) |
| 15 | # mmu05200 Pathways in cancer - (12) | # mmu04670 Leukocyte transendothelial migration - (10) | # hsa03010 Ribosome - (7) | mmu05215 Prostate cancer (4) |
| 16 | # mmu00010 Glycolysis / Gluconeogenesis - (11) | # mmu05414 Dilated cardiomyopathy - (9) | # hsa04010 MAPK signaling pathway - (7) | mmu05016 Huntington's disease (4) |
| 17 | # mmu00230 Purine metabolism - (11) | # mmu05412 Arrhythmogenic right ventricular cardiomyopathy - (9) | # hsa04670 Leukocyte transendothelial migration - (7) | mmu00970 Aminoacyl-tRNA biosynthesis (4) |
| 18 | # mmu04910 Insulin signaling pathway - (10) | # mmu00620 Pyruvate metabolism - (9) | # hsa04810 Regulation of actin cytoskeleton - (6) | mmu04612 Antigen processing and presentation (4) |
| 19 | # mmu00020 Citrate cycle (TCA cycle) - (10) | # mmu05410 Hypertrophic cardiomyopathy (HCM) - (9) | # hsa04114 Oocyte meiosis - (6) | mmu00190 Oxidative phosphorylation (3) |
| 20 | # mmu04612 Antigen processing and presentation - (10) | # mmu04520 Adherens junction - (8) | # hsa00620 Pyruvate metabolism - (6) | mmu04110 Cell cycle (3) |
| 21 | # mmu04540 Gap junction - (10) | # mmu04260 Cardiac muscle contraction (8) | # hsa00970 Aminoacyl-tRNA biosynthesis - (6) | mmu04010 MAPK signaling pathway (3) |
| 22 | # mmu04670 Leukocyte transendothelial migration - (10) | # mmu05200 Pathways in cancer - (7) | # hsa04612 Antigen processing and presentation - (6) | mmu04722 Neurotrophin signaling pathway (3) |
| 23 | # mmu04010 MAPK signaling pathway - (9) | # mmu03040 Spliceosome - (7) | **# hsa04144 Endocytosis - (6)** | mmu00620 Pyruvate metabolism (3) |
| 24 | # mmu04520 Adherens junction - (9) | # mmu04910 Insulin signaling pathway - (7) | # hsa04910 Insulin signaling pathway - (5) | mmu03040 Spliceosome (3) |
| 25 | **# mmu04144 Endocytosis - (8)** | **# mmu04020 Calcium signaling pathway - (7)** | # hsa03030 DNA replication - (5) | mmu04146 Peroxisome (3) |
| 26 | mmu05412 Arrhythmogenic right ventricular cardiomyopathy - (8) | # mmu04540 Gap junction - (6) | # hsa00020 Citrate cycle (TCA cycle) - (5) | mmu00230 Purine metabolism (3) |
| 27 | # mmu05416 Viral myocarditis - (7) | # mmu04110 Cell cycle - (6) | # hsa05012 Parkinson's disease - (5) | mmu05010 Alzheimer's disease (3) |
| 28 | # mmu00480 Glutathione metabolism - (7) | # mmu04722 Neurotrophin signaling pathway - (6) | # hsa00250 Alanine, aspartate and glutamate metabolism - (5) | mmu03018 RNA degradation (3) |
| 29 | # mmu04666 Fc gamma R-mediated phagocytosis (7) | # mmu00230 Purine metabolism - (6) | # hsa04120 Ubiquitin mediated proteolysis (4) | mmu04621 NOD-like receptor signaling pathway (3) |
| 30 | # mmu04120 Ubiquitin mediated proteolysis (7) | **# mmu04144 Endocytosis (6)** | # hsa05010 Alzheimer's disease – (4) | mmu05213 Endometrial cancer (2) |
